# Implementation of an Antibiotic Therapy Protocol for Open Fractures in the Emergency Department

**DOI:** 10.51894/001c.6898

**Published:** 2018-09-26

**Authors:** Terrence Endres, Kristopher Danielson, Stephen O’Neil, Shawn Brandenburg, Teresa Hall, Hunter Ross

**Affiliations:** 1 Metro Health Orthopaedic Traumatologist, Orthopaedic Associates of Michigan; 2 Metro Health Orthopaedic Surgery Resident

**Keywords:** antibiotics, emergency department trauma, orthopedics, open fracture

## Abstract

**CONTEXT:**

Well established in the Emergency Department (ED) literature is that the most important factor in decreasing subsequent infection rate in open fractures is the time to first administration of antibiotics. As such, the authors developed a new ED open fracture antibiotic protocol to facilitate more expeditious antibiotic administration and appropriate choice of antibiotics.

**METHODS:**

During Phase 1 of this project, the authors identified the 2012 - 2016 historical length of time from presentation of an open fracture to the possible initiation of antibiotic therapy at their institution. Results demonstrated critical areas for improvement in both timing and types of antibiotics administered. Phase 2 of the study evaluated the effect of the new open fracture antibiotic protocol. Sample cases from both phases were then further identified based on type of open fracture, time to initiation of antibiotics from ED presentation, type of antibiotics, and time to definitive treatment. Analyses were performed using GraphPad proprietary software.

**RESULTS:**

A random sample of 110 patients were included from Phase 1 and 27 patients from Phase 2. A total of 43 Phase 1 patients were administered cefazolin (Kefzol, Ancef); the remainder of the patients received a number of different antibiotics. During Phase 2, all 27 patients received cefazolin and Gentamycin if necessary per the new protocol. The average time to initiation of antibiotics was 0.907 hours during Phase 1 compared to 0.568 hours in Phase 2. The new protocol also significantly decreased the average time to antibiotics in ED from 2.17 hours to 1.82 hours when including EMS transfer time. Average time to definitive treatment in the operating room was 6.63 hours during Phase 1 and was significantly lowered to 3.97 hours during Phase 2.

**CONCLUSIONS:**

Timing to initiation of antibiotics after open fractures is the most important aspect to decrease infection rates. In order to decrease these times, the authors implemented a new ED protocol that specifically stated the type of antibiotic to be given based on the open fracture without orthopedics needing to be notified before administration. Ideally, the use of such protocols in ED settings will serve to greatly decrease infection risks after open fracture.

## INTRODUCTION

A recent upgrade to a certified Level 2 trauma center at the author’s institution (Michigan-based Metro Health) accentuated emergency department (ED) providers’ awareness of the importance of high quality care for their trauma patients. A Level 2 trauma center hospital is able to initiate definitive care for all injured patients and includes 24-hour coverage by the specialties of orthopedic surgery, trauma surgery, neurosurgery, anesthesia, emergency medicine and critical care.^1^ One common type of traumatic injury seen in ED settings is an open or “compound” bone fracture in which there is also an open wound or break in the skin.

It has been established in the medical literature that the most important factor to decrease later infection rates in open fractures is the time to administration of antibiotic therapy.^2^ Until 150 years ago, an open fracture was associated with high morbidity and often resulted in amputation.^2^ Despite improvements in sanitization and hemorrhage, mortality rates continued to be high. An early 1881 study by Billroth noted that more than half of the patients in a study with 93 open fractures died due to sepsis.^2^

As medical research continued, the advances by Pasteur, Koch and Lister noted large advancements in patient survival outcomes. They advocated for limb splinting and wound extension with excision and debridement procedures that yielded favorable outcomes. There has continued to be improvement and better outcome prediction with open fracture management and notable advancements as it pertains to successful fracture reduction and fixation along with serial irrigation and debridements.^3–6^

With an improved understanding of the optimal treatment of open fractures, a way to categorize them was needed. A fracture classification defined by Gustilo and Anderson ^3^ was one of the first and remains most widely accepted. (Figure 1) According to this framework, open fractures are classified by Types I-III, with Type III being further classified based on soft tissue coverage and degree of vascular compromise.

**Figure:**
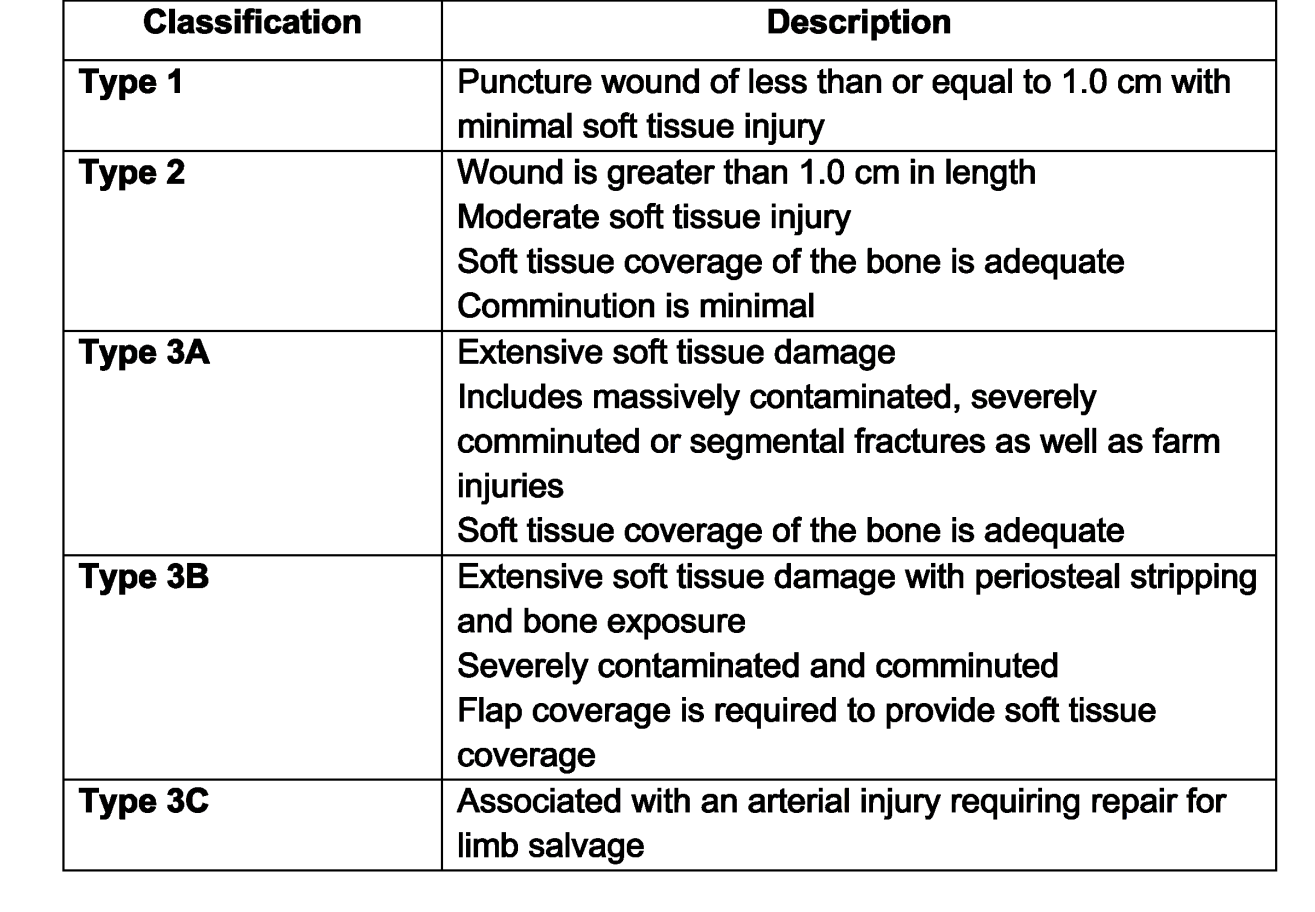
Figure 1 Gustilo-Anderson Open Fracture classification

In 1976, Gustilo and Anderson reported a series of 673 open fractures of long bones treated from 1955 to 1968 with infection rates varying between 12% (1955-1960) and 5% (1961-1968). In this group’s later 1984 prospective study, 352 patients were managed as follows: debridement and copious irrigation, primary closure for Type I and II fractures and secondary closure for Type III fractures, no primary internal fixation except in the presence of associated vascular injuries, cultures of all wounds, and oxacillin-ampicillin before surgery and for three days postoperatively.^4^ To compare the two periods, the infection rates were 44% in the first retrospective study ^3^ and 9% in the later prospective study ^4^ for Type III open fractures (severe soft-tissue injury, segmental fracture, or traumatic amputation).

The recent literature on open fracture management has continued to reinforce that the most important variable for open fracture management is timely administration of antibiotic therapy. In their 1989 article, Patzakis and Wilkins reported an infection rate of 4.7% when antibiotics were administered within three hours of open fracture injury, compared to 7.4% when the treatment was delayed.^5^

### Purpose of Project

The goal of this quality improvement project was to examine the relative effects of an ED antibiotic administration protocol for open fracture patients at the authors’ institution. Specific objectives were to evaluate the effect of the new protocol to decrease the time to first antibiotic administration, and standardize the type of antibiotics administered for open fracture patients.

## METHODS

IRB approval had been obtained from the author’s institution before data collection was begun and the authors followed the PDSA model (Plan, Do, Study, Act) to plan the project.^6^ More specifically, Phase 1 consisted of the “Plan” and “Do” aspects of the model, while Phase 2 consisted of the “Study” and “Act” aspects of the model.

During Phase 1, the authors completed a retrospective chart review using ICD-9 codes ^7^ (all open fracture ICD-9 codes used during an EHR search) to isolate open fracture diagnosis patients in the ED from a certain time period. Inclusion criteria included ICD-9 code for open fracture from December 2012 through December 2016. Exclusion criteria included evaluation and treatment at another institution for the same fracture prior to arrival. Statistical analyses were performed by a local campus-based statistician (see acknowledgements) using Graph Pad proprietary software.^8^ Phase 1 results demonstrated critical areas for improvement in both decreasing time to antibiotic therapy as well as consistency among antibiotic choices in the setting of open fractures. With these results, a new antibiotic administration protocol for open fractures was developed before Phase 2 and put on display in the authors ED, with the new protocol also incorporated into the institution’s electronic health record (EHR).

### New Antibiotic Administration Protocol

The new antibiotic administration protocol for open fractures worked as follows. If a patient arrived to the ED with an open fracture, the ED attending physician typed open fracture into orders in the Epic EHR chart. A list then appeared so that the attending physician could simply click on the type of open fracture and from there indicated antibiotics were pre chosen. For example, if a patient came in with a fracture with puncture wound (wound less than 1.0 centimeter (cm) associated with it, then the ED attending typed in open fracture and clicked on the choice for puncture wound (wound less than 1.0 cm): 2 grams (gm) cefazolin (Kefzol, Ancef), and the antibiotic was immediately administered.

If the wound appeared to be greater than 1.0 cm, then the ED attending clicked that option and Gentamycin 5mg/kg was added to the patient’s antibiotic regimen to ensure proper gram-positive and gram-negative bacterial coverage. If the injury was a suspected farm injury, Penicillin G was added to the regimen without the ED attending having to distinguish between contaminated or clean wounds. This also eliminates the need for the ED attending physician to differentiate between the types of Gustilo’s classification of open fractures and still provide adequate antibiotic coverage. If the patient was allergic to Penicillin, Vancomycin 15mg/kg (dosage per weight) could have been administered as an alternative (Figure 2).

**Figure:**
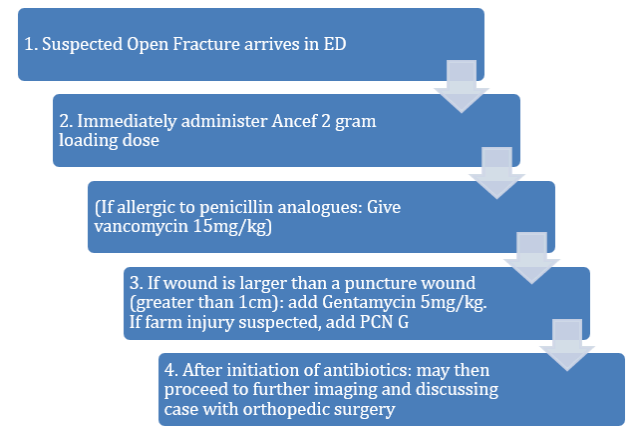
Figure 2 Algorithm for Open Fracture Antibiotic Initiation in the Emergency Department

Frequently, patient weight was not obtained in a timely fashion in a trauma code, therefore instead of using weight-based dosing for cefazolin, a dose of 2 gm was chosen for simplification of antibiotic dosing for expeditious administration. This study excluded pediatric open fractures and therefore pediatric dosing of antibiotics.

Finally, during Phase 2 a second review was performed one year after implementation of the new antibiotic protocol. The charts of eligible sample patients were randomly chosen based on project exclusion and inclusion criteria. Inclusion criteria included ICD-9 code ^7^ for open fracture from December 2016 to December 2017. Exclusion criteria included evaluation and treatment at another institution for the same fracture prior to arrival. The authors further evaluated cases based on type of open injury per the Gustilo/Anderson classification, Type 1, 2, or 3; time to initiation of antibiotics from ED presentation; type of antibiotics; and time to definitive surgical treatment.

## RESULTS

### Phase 1: December 2012-December 2016 (before new protocol)

Of the charts identified per the inclusion criteria, a total of 118 patients were included in analyses. Of those 118, eight (6.8%) were miscoded as an open fracture, leaving 110 patients included in the retrospective review. Of the 110 fractures, 25 (22.7%) were Gustilo Type 1; 36 (32.7%) were Gustilo Type 2, and 12 (11.0%) were coded as Gustilo Type 3; 9 (8.2%) as Type 3A and 1 (0.09%) as Type 3B, and 2 (1.8%) as Type 3C. The remaining 37 (33.6%) were coded as open fractures with no distinction.

In addition, the types of antibiotics administered were also recorded; 43 (75.4%) patients were administered cefazolin 1 gm intravenous (IV); two (1.8%) patients were administered cefazolin 2 gm IV; two (1.8%) patients were administered cefazolin 1 gm intramuscularly; 19 (17.3%) patients were administered Ancef 1 gm IV and Gentamycin 250 mg IV; one (0.09%) patient was administered Augmentin orally; and one (0.09%) patient was administered Ceftriaxone 1 gm IV. The other combinations of antibiotics that were administered during Phase 1 are listed in Table 1.

**Table:** Table 1 Types of Antibiotics Prescribed During Phase 1

**Phase 1: Antibiotics Administered in ED**	
**Antibiotic**	**# of Patients**
Ancef 1 gram IV	(75.4%) 43
Ancef 2 gram IV	2
Ancef 1 gram IM	2
Ancef 1 gram IV and Gentamycin 250 mg IV	19
Augmentin PO	1
Ceftriaxone 1 gram IV	1
Clindamycin 600 mg IV	1
Keflex 500 mg PO QID	1
Rocephin 1 gram IV	3
Vancomycin 2 grams IV	1

Patients’ average length of hospital stay was reported as 2.63 (SD 4.082) days. Average time to definitive treatment in the operating room from initial ED presentation was 6.63 hours ranging from one to 26 hours. The average time to initiation of antibiotic administration for open fractures after ED presentation was 0.907 hours, ranging from 0.10 to 3.10 hours. These time estimates did not include time from injury to presentation to the ED.

According to the National Emergency Medical Services Database, response time to scene is 14 minutes on average; scene time is 40 minutes on average; transport time is 22 minutes on average. This totals 76 minutes on average.^9^ This additional time should be considered by providers in the ED when assessing open fractures and an accurate time from injury to initiation of antibiotics should be documented.

### Phase 2: December 2016-December 2017 (after protocol)

During this phase, 35 eligible patients were identified. Of those 35, eight (22.8%) were miscoded as an open fracture, leaving a total of 27 patients included in the retrospective review. Of these 27 fracture patients, 11 (40.7%) were Gustilo Type 1; seven (26.0%) were Gustilo Type 2 and nine (33.3%) were coded as Gustilo Type 3 (eight as Type 3A, zero as Type 3B, and one as Type 3C).

Data concerning the type of antibiotics administered as a deviation from current protocol was also collected. Twenty five (93.0%) of Phase 2 patients were administered cefazolin IV plus Gentamycin, if appropriate, per protocol. Two patients were considered outliers, one patient who was administered Unasyn and one patient who received Zosyn. Of these two patients who were not administered cefazolin, the ED staff switched them to cefazolin per protocol within one and two hours respectively, therefore they met criteria of the phase 2 protocol. Therefore, 27 (100%) of Phase 2 patients received appropriate antibiotic treatment. (Table 2) This is compared to Phase 1, which was before the new antibiotic protocol for open fractures was in effect, in which appropriate antibiotic use was only seen in 66 of 74 (89%) of patients (p > 0.05). (Table 1)

**Table:** Table 2 Types of Antibiotics Prescribed during Phase 2

**Phase 2: Antibiotics Administered in ED**		
**Gustilo Classification**	**Antibiotics Administered**	**# of Patients**
Type 1	Ancef IV	11
Type 2	Ancef plus Gentamycin IV	7
Type 3	Ancef plus Gentamycin IV	9

No farm injuries during Phase 2.

Length of stay in the hospital averaged 4.9 days in Phase 2 compared to 2.63 days during Phase 1. This finding could be explained by variations in the severity of the fractures and need for multiple procedures. Average time to definitive surgical treatment from initial ED presentation was 3.97 hours (SD 2.506) during Phase 2 compared to 6.63 hours (SD 5.490) during Phase 1 (p < 0.05), ranging from 1.30 hour to 8.43 hours. Also important to note is that no Phase 2 sample patients waited longer than four hours for definitive surgical treatment (Graph 1), although the authors did not attempt to correlate this measure with initiation of timing to antibiotic administration.

**Figure:**
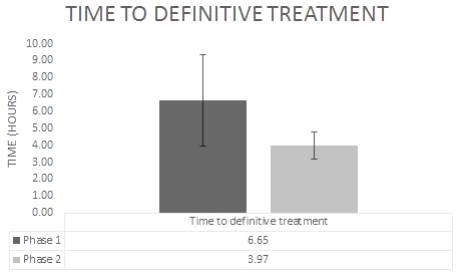
Graph 1 Time from Arrival to Definitive Fixation in the Operating Room

In Phase 2, the average time to initiation of antibiotics for open fractures after ED presentation was 0.568 hours (34 minutes) (SD 0.4556), ranging from 0.1 to 2.2 hours (6 minutes to 132 minutes). This is compared to Phase 1, which was 0.907 hours (54.4 minutes) (SD 0.7356) ranging from 0.1 to 3.1 hours (6 minutes to 186 minutes) (p < 0.05) (Graph 2).

**Figure:**
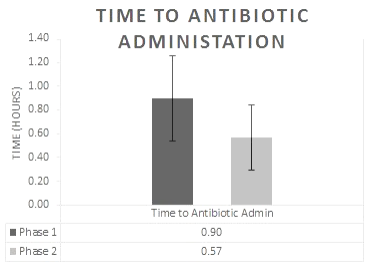
Graph 2 Time from Arrival to ED to Antibiotic Administration

## DISCUSSION

The initiation of antibiotic therapy after an open fracture has been proven to be the most important factor in preventing perioperative infection.^2^ Patzakis et al (2000) conducted a prospective study that showed a statistically significant reduction in infection rates attributed to the administration of ciprofloxacin compared with no antibiotic administration after open fractures.^10^ Additionally, this group found a considerable reduction in infection rates when antibiotics were administered less than three hours after injury compared with longer than three hours after injury (4.7% versus 7.4%).^5^

Similarly, Lack and colleagues (2015) also reported that time to antibiotic administration was predictive of infection.^11^ Cefazolin was the only agent given in 93.4% of cases. The overall deep infection rate was 17.5%. Patients who received antibiotics within one hour of injury had a 6.8% infection rate compared with 27.9% in those receiving antibiotics after 90 minutes.^11^

One of the shortcomings of the Gustilo and Anderson classification ^3^ includes lack of inter-observer reliability. In fact, Brumback and Jones (1994) recommended delaying fracture classification until the first operative debridement.^12^ As more data suggest that time to debridement is not predictive of infection, the average time to debridement is likely to increase. If time to debridement increases, then time to classification and appropriate antibiotic treatment could be delayed.^12^ In addition, since the importance of the time to antibiotic administration with effective gram-negative coverage has not yet been well established, we used this possible complication to adjust our specific open fracture antibiotic protocol.

It was very important to the authors that after implementation of our Phase 2 protocol, all patients received antibiotic therapy within three hours (100% compliance) from time of injury. Based on the results of Patzakis et al.^5,10^ along with the results observed in this study (Graph 2), suggests that our Phase 2 patients were at decreased risk for infection when compared to the relatively delayed time to antibiotics administration during Phase 1.

Finally, the initial Phase 1 data clearly demonstrated there was no uniform protocol for specific antibiotic used in the ED at this institution. Although a majority of sample patients treated before the protocol received cefazolin 1 gm IV (75.4%), there were still roughly 25% of patients who received a variety of antibiotics. After the initiation of the protocol, all open fractures were appropriately administered cefazolin and/or Gentamycin.

According to Sirkin, for low-grade (I and II) open fractures, antibiotics should be directed at mainly gram-positive and some gram-negative coverage.^13^ A first-generation cephalosporin, such as cefazolin, should be used. A 2-gm loading dose is given, followed by 1 gm every eight hours. For the higher-grade fractures (III-A and III-B), the treating physician must worry more about gram-negative coverage. An aminoglycoside (Gentamycin or Tobramycin) must be added to help prevent these types of infections.^13^

For grade III-C fractures (e.g., limbs with poor vascular status and farm injuries), the presence of anaerobes is more likely. In addition to a cefazolin and Gentamycin, aqueous Penicillin G 4 million units every four hours should be added. The duration of antibiotic therapy has historically been seven to 10 days.^4^ Most likely this was recommended to simulate the duration of wound healing. Although definitive evidence is not presently available, current recommendations for uncomplicated, grade I or II fractures is antibiotic coverage for 24 to 48 hours after wound closure. For grade III fractures, this should be extended to 48 to 72 hours after definitive wound closure.^4^

When evaluating the data from Phase 2 of this study, we recognize that some patients may not have been appropriately administered tetanus toxoid or immunoglobulin according to Centers for Disease Control (CDC) recommendations. If a patient has had more than a 10-year lapse in a tetanus booster or if a patient is immunocompromised they should be administered both the toxoid and the immune globulin (250 – 500 IU)^14^ The authors have noted this would be an easy future additional element for the protocol based on the patient’s immunization status.

Furthermore, this study reinforces the tenets of osteopathic medicine. Providers should consider that an open fracture is not just an isolated event, but an inciting event that can have further health repercussions when not addressed in a timely and appropriate manner. Potential repercussions include further need for surgical intervention, osteomyelitis, bacteremia, sepsis and even death. As demonstrated in osteopathic principles, this highlights the principle that the body is a unit and the interrelationship of structure and function. By treating open fractures with appropriate antibiotics as soon as possible, physicians can decrease the risk of patients developing such adverse events.

## CONCLUSIONS

In summary, timing to initiation of antibiotics after open fractures has been shown to be an important aspect to decrease infection rates in multiple prospective studies. As previously stated, the 2015 Lack study found lower incidence of infection in grade III injuries if antibiotics were administered less than 66 minutes after injury.^11^ However, most experts recommend less than three hours post-injury for the majority of open fractures as the ideal goal for initiation of antibiotics.^2^ The type of antibiotics given has also been proven in multiple articles.

At the author’s specific institution, the average time to initiation of antibiotics had been about 2.17 hours from time of injury with some patients waiting as much as 4.36 hours, prior to surgical intervention. In response, a new protocol for administration of clinically indicated antibiotics for open fractures in the ED was implemented at the author’s institution in December 2016, leading to a decrease in time to initiation of antibiotics.

The further development and testing of open fracture protocols can decrease provider confusion and discrepancies among attending ED physicians and residents. In further testing, the incorporation of additional key elements such as tetanus toxoid being added to the protocol and standardized antibiotic prescribing practices can serve to improve care in this complex aspect of orthopedic trauma care.

### Conflict of Interest

The authors declare no conflict of interest.
